# Potential lipid-lowering effects of *Coffea arabica* pulp extract product in hyperlipidemia-obese subjects: a randomized double-blind placebo-controlled trial

**DOI:** 10.3389/fnut.2026.1755054

**Published:** 2026-03-12

**Authors:** Supawan Buranapin, Atcharaporn Ontawong, Chutima S. Vaddhanaphuti, Piti Inthaphan, Jakkapong Inchai, Thanthakan Saithong, Kanjana Narkprasom, Varunya Fuangchoom, Doungporn Amornlerdpison

**Affiliations:** 1 Section of Endocrinology and Metabolism, Department of Medicine, Faculty of Medicine, Chiang Mai University, Chiang Mai, Thailand; 2 Division of Physiology, School of Medical Sciences, University of Phayao, Phayao, Thailand; 3 Innovative Research Unit of Epithelial Transport and Regulation (iETR), Department of Physiology, Faculty of Medicine, Chiang Mai University, Chiang Mai, Thailand; 4 Faculty of Engineering and Agro-Industry, Maejo University, Chiang Mai, Thailand; 5 Graduate Program in Food Engineering, Faculty of Engineering and Agro-Industry, Maejo University, Chiang Mai, Thailand; 6 Center of Excellence in Agricultural Innovation for Graduate Entrepreneur, Maejo University, Chiang Mai, Thailand; 7 Faculty of Fisheries Technology and Aquatic Resources, Maejo University, Chiang Mai, Thailand

**Keywords:** coffee pulp aqueous extract, CPE product, hyperlipidemia, lipid profiles, obese

## Abstract

**Background:**

*Coffea arabica* pulp aqueous extract (CPE) exhibits anti-hyperlipidemia, anti-hyperglycemia, and anti-inflammatory effects *in vitro* and *in vivo*. This randomized, double-blind, controlled study evaluated the lipid-lowering effects of the CPE product in hyperlipemia-obese participants.

**Methods:**

Seventy-nine obese men and women [BMI ≥ 25 kg/m^2^ and low-density lipoprotein cholesterol (LDL-C) ≥ 130 mg/dL] were randomly allocated to the CPE product and control groups. The CPE product or placebo was consumed twice daily for 24 weeks. The lipid profiles, BW, BMI, fat mass, and diabetic diagnosis parameters were assessed alongside adverse events measurements.

**Results:**

Within-group analysis showed a significant reduction of LDL-C, triglyceride (TG), and total cholesterol (TC) levels by 14.9, 18.9, and 8.0%, respectively, while increasing high-density lipoprotein cholesterol (HDL-C) levels by 6.0% in the CPE product group compared to baseline after 24 weeks of intervention. In the placebo group, LDL-C and HDL-C levels decreased significantly by 7.7 and 5.3%, respectively, at week 24, but TC and TG levels did not change significantly. After adjustment for screening lipid values, the CPE group showed greater reductions in total cholesterol, triglycerides, and LDL-C than the placebo group (−6.3% vs. −2.4%, *p* = 0.048; −12.5% vs. +1.17%, *p* = 0.078; and −13.7% vs. −7.1%, *p* = 0.01, respectively). HDL-C increased in the CPE group but decreased in the placebo group after adjustment (+7.1% vs. −5.0%, *p* < 0.0001). The adverse events were not different between the experimental groups.

**Conclusion:**

This study suggests that the CPE product could be a nutraceutical product option for lowering lipids in hyperlipidemic patients. However, this study has limitations, including the short treatment period, the small number of enrolled participants, and its exploratory nature.

**Clinical trail registration:**

This study was registered at the Thai Clinical Trial Registry with the identification number 20241117005. https://www.thaiclinicaltrials.org/.

## Graphical Abstract

**Figure:**
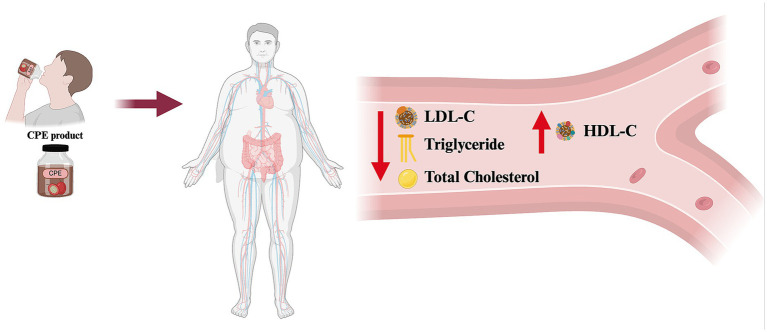

## 1 Introduction

Nowadays, obesity has become a global problem due not only to a sedentary lifestyle, but also to poor diets and/or excessive consumption of ultra-processed foods. World Health Organization (WHO) data show that worldwide obesity in adults has more than doubled since 1990. In 2022, 43% of adults aged 18 years and over were overweight, and 16% were living with obesity. WHO reported in June 2021 that at least 2.8 million individuals worldwide die each year from obesity-related causes, indicating that the disease has epidemic proportions (1). Obese adults are found to have combined dyslipidemia characterized by moderate to severe increases in triglycerides (TG) and non-high-density lipoprotein cholesterol (non-HDL-C) with decreased high-density lipoprotein cholesterol (HDL-C) (2, 3). It is associated with chronic low-grade inflammation in various organs such as adipose tissue, liver, pancreas, and brain (4, 5). Obesity is strongly correlated with a higher risk of cardiovascular disease (CVD) death, especially coronary heart disease and ischemic stroke (3, 6, 7). Furthermore, it is also a known risk factor for developing metabolic syndrome, including hyperglycemia, hyperlipidemia, and hypertension, which are high-risk factors for type 2 diabetes mellitus, non-alcoholic fatty liver disease, and atherosclerotic CVD (8, 9). Cardiovascular risk is strongly associated with low-density lipoprotein cholesterol (LDL-C) levels, and there is cardiovascular benefit from lipid-lowering therapies that reduce LDL-C levels (10). Previous studies reported that lowering LDL-C levels had benefits in reducing major cardiovascular events; for instance, every 39 mg/dL of LDL-C level reduction with statin therapy would reduce major cardiovascular events by 16% (11, 12). Thus, any strategy for the prevention of risk factors for obesity might help to reduce its complications and death rates. Currently, there are various approaches for weight reduction, and the use of natural dietary supplements has become an increasingly popular option. Therefore, this study focuses on natural products to minimize potential side effects.

**Figure 1:**
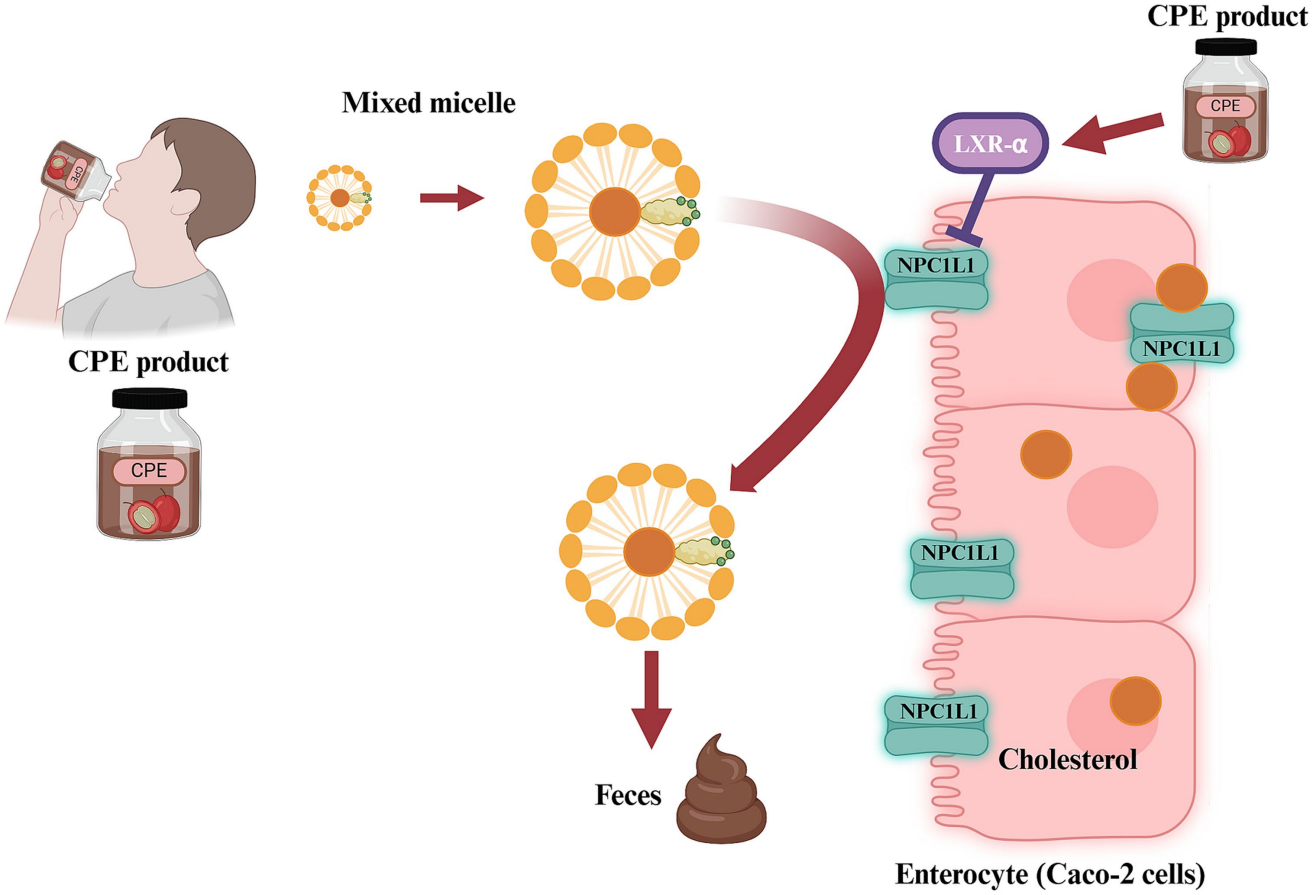
The proposed mechanism of action of *Coffea arabica* pulp extract (CPE) product.

Coffee processing generates a large amount of coffee by-products and residues from coffee cherry to cup, causing critical environmental issues, including water and land pollution (13, 14). Among these, coffee pulp (CP) waste constituted 29% of the dry weight and 41% of the wet weight of the cherries (13, 15). CP is enriched in fibers, sugars, proteins, tannins, and phenolic compounds (16). Analysis of CP revealed several phenolic compounds, with chlorogenic acid (CGA) being the most abundant at approximately 2.6%, whereas caffeine was at 1.6% (16, 17). CGA has been demonstrated to have several biological activities, such as anti-inflammation, anti-oxidative, anti-hyperglycemic, and anti-hyperlipidemic effects (18–28). In addition, CP-rich CGA exhibited high antioxidant activity against 1,1-diphenyl-2-picrylhydrazyl (DPPH) radical and superoxide anion (21, 23). Previous study reported that CP extract (CPE) inhibited intestinal cholesterol absorption in human colon carcinoma (Caco-2) cells (Figure 1), in an *ex vivo* jejunal epithelial loop, and *in vivo* (29). Furthermore, CPE reduced plasma lipid profile, improved insulin sensitivity, and hepatic lipid accumulation in high-fat diet-induced hepatic steatosis and hepatocellular carcinoma (HepG2) cells, respectively (30, 31). CPE also exerted anti-inflammation by suppressing pro-inflammatory cytokines and inactivating nuclear factor-kappa B (NF-κB) and mitogen-activated protein kinase (MAPK) signaling pathways in macrophage cells (32). Taken together, CPE has the potential to be an optional nutraceutical product for hyperlipidemia-induced hepatic steatosis and inflammation. To address the potential use of CPE in humans, therefore, this study aimed to determine the lipid-lowering effects of CPE product on lipid profiles in hyperlipidemia-obese participants. The primary objective of this study was to compare the change in each lipid profile and inflammatory markers from baseline to week 24 and compare the changes in lipid profiles and inflammatory markers between the CPE product and placebo. The secondary objectives were to evaluate the effect of the CPE product on body weight (BW), fat mass, body mass index (BMI), waist circumference, fasting blood sugar (FBS), hemoglobin A1C (HbA1C), and insulin resistance in hyperlipemia-obese participants. The prespecified safety analysis included serious adverse events (SAEs), adverse events that led participants to discontinue the trial treatment, and common AEs after consuming CPE product. Unlike previous studies that mainly investigated CGA or coffee extract in healthy or mixed populations with relatively short intervention periods, the current study is one of the few randomized controlled trials specifically conducted in obese individuals with hypercholesterolemia over a 24-week intervention.

## 2 Materials and methods

### 2.1 Preparation of a CPE product and placebo for clinical assessment

CPE product was kindly provided by Hillkoff Co., Ltd. (Chiang Mai, Thailand). Microorganisms were eliminated by heating the product at 95–100 °C for 24 h using far-infrared (FIR) heating to preserve its key bioactive compounds. After drying, the coffee pulp was stored in oxygen-impermeable bags with a low oxygen transmission rate (OTR) to prevent oxidation. These processes are necessary to maintain the organoleptic quality of the CPE product. In addition, the placebo was prepared in identical, sealed bottles of the same appearance.

### 2.2 Quality control of CPE product and placebo

The active ingredients of the CPE product, CGA (Sigma-Aldrich, St. Louis, MO, United States), and caffeine (Sigma-Aldrich, St. Louis, MO, United States) were used as a reference for validation and quantitation by high-performance liquid chromatography (HPLC) with diode array detection on Agilent 1,200 series (LabX, Midland, Canada) according to the ISO/IEC 17025 method (33) at the Science and Technology Service Center, Faculty of Science, Chiang Mai University, Chiang Mai, Thailand. The HPLC separation was performed on an Eclipse XDB-C18 (4.6×150 mm, 5 μm) using a binary solvent system, mobile phase solvent A (2% acetic acid dissolved in HPLC water) and mobile phase solvent B (absolute methanol). Separation was performed using a linear gradient elution as follows: 12% A for the first 0–5 min; 12–15% A from 0 to 5 min; 15–30% A from 5 to 15 min; held isocratically at 30% A from 15 to 19 min; and increased from 30 to 50% A from 19 to 27 min. at a flow rate of 0.5 mL/min with detection at 280 nm. CGA and caffeine found in the CPE product and the placebo were detected relative to the corresponding peaks of references, and the concentration of each component was subsequently quantified by peak area under the curve relative to the reference compound, CGA and caffeine, in triplicate.

### 2.3 Determination of total polyphenols in CPE product and placebo

The total phenolic content of the CPE product and placebo was evaluated in triplicate using the UV–Vis spectrophotometer (34) at the STSC-CMU, Chiang Mai, Thailand. Total polyphenol content was quantified based on a gallic acid standard curve and expressed as mg/L of gallic acid equivalents (GAE).

### 2.4 Determination of antioxidant capacity

The CPE product and the placebo were assessed for their scavenging abilities against DPPH and ABTS+• radicals as previously reported (35, 36). Briefly, the CPE product or placebo range of 5–100% V/V was combined with 7 mM ABTS^+•^ reagent, and the absorbance was monitored at 734 nm for 6 min using a SynergyTM HT microplate reader (Biotek, VT, United States). Vitamin C was used as a positive control. A concentration-response curve was used to calculate the data, and the radical scavenging activity was measured as the effective concentration at which the ABTS^+•^ radical was scavenged. Likewise, 100 μM DPPH solution was combined with either the CPE product or the placebo, and the mixture was incubated for 30 min at room temperature in the dark. The amount of residual DPPH was measured at 517 nm.

### 2.5 Determination of cholesterol micelle morphology using scanning electron micrograph

The cholesterol micelle components: 3 mM unlabeled cholesterol, 6 mM sodium taurocholate, and 0.15 mM phosphatidylcholine were mixed in phosphate-buffered saline (PBS) and dried under N_2_ to form cholesterol micelles. The cholesterol micelle was reconstituted in PBS by sonication for 1 h. The solution was filtered through a 0.2-μm syringe filter membrane. CPE product and placebo were added to the filtrate at a 1:10 ratio and incubated at 37 °C for 3 h. The CPE product or placebo incorporated cholesterol micelles was added to the copper tape-covered stub and air-dried for 3 days. After that, the incorporated cholesterol micelles were coated with Au^+^, and the images were taken with a scanning electron microscope (SEM) JEM-IT800 (Field emission) under 200 kv at the Advanced Scientific Instrument Unit (ASci), Faculty of Science, Chiang Mai University.

### 2.6 Determination of cholesterol micellar size and micellar complex solubility

The cholesterol micellar size was measured as previously published (29). Micelles were prepared as in a previous study. CPE product and placebo were added to the mixed micelle solution, which was incubated for 3 h at 37 degrees Celsius. Finally, the size of the micellar complex was determined with a particle size analyzer (Malvern Instruments Ltd., Malvern, United Kingdom).

The micellar complex solubility was evaluated as described in our previous study (29). In short, 1 mM unlabeled cholesterol, 1 mM sodium taurocholate, and 0.6 mM phosphatidylcholine were combined with the micelle solution in PBS. The solution of mixed micelles was incubated for 3 h at 37 °C with the CPE product and placebo. A 0.22 μm membrane was used to filter the lipid micelles to isolate the intermicellar complex from the precipitated micellar. A commercial colorimetric cholesterol test (Biotechnical Co., Ltd., Bangkok, Thailand) was used to measure the filtrated micellar cholesterol.

### 2.7 Bile acid binding capacity

Bile acid binding capacity was examined as previously reported (29). Briefly, CPE product, placebo, and cholestyramine (1 mg/mL), a bile acid sequestrant, were incubated for 2 h at pH 7.0, 37 °C, with or without 2 mM taurocholic acid, glycodeoxycholic acid, or taurodeoxycholic acid. To separate bound and free bile acids, the bile acid mixture was centrifuged for 10 min at 10,000 rpm, and the supernatant was filtered through a 0.22 μm membrane filter. A reaction mixture comprising 0.133 M tris-buffer at pH 9.5, 1 M hydrazine hydrate, 7.7 mM nicotinamide adenine dinucleotide (NAD), and 1 unit/mL 3α-hydroxysteroid dehydrogenase was combined with the filtrated free bile acid. The reaction mixture was incubated for an additional 2 h at 37 °C. At 340 nm, the rate of thio-NADH production was measured and expressed as a percentage of the control.

### 2.8 Participants

Eligible participants aged 18 years or older were recruited at Chiang Mai University Hospital, Chiang Mai, Thailand, with a BMI ≥ 25 kg/m^2^ and LDL-C ≥ 130 mg/dL. Participants were required not to take any lipid-lowering therapy unless they had taken lipid-lowering agents at a stable dose for at least 8 weeks before screening. Reproductive-age female participants were required to use contraception during the entire trial period. Postmenopausal women or sterilized female participants were eligible for inclusion. Additionally, participants were required to attend throughout the 6 months of the study. All the trial participants provided written informed consent prior to participation. Exclusion criteria were any types of diabetes mellitus, renal impairment (estimated glomerular filtration rate; eGFR below 45 mL/min/1.73 m^2^), hepatic impairment (aspartate transaminase; AST or alanine transaminase; ALT > 3 fold of upper normal limit (UNL), total bilirubin > 2 mg/dL, direct bilirubin > 1 mg/dL), uncontrolled thyroid disease, post-organ transplantation, immunodeficiency state (taking any immune suppressive agents or active malignancy or human immunodeficiency virus (HIV) infection), pregnancy, lactating women, taking drugs which induced hyperglycemia (corticosteroids), recently attended in other trial within 8 weeks, taking drugs which might affect lipid profiles (oral contraceptive pills, high dose diuretics, beta-blockers, antiretroviral protease inhibitors, carbamazepine, anabolic steroids) except it was used for a stable dose at least 8 weeks, taking weight-lowering medications within 3 months, coffee or caffeine allergy, triglyceride level > 400 mg/dL, previous hospitalization within 2 months, drinking alcohol more than 2 times per week (each time for >1 standard drink for female or >2 standard drinks for male), abnormal gastrointestinal symptoms (nausea, vomiting, anorexia, early satiety, diarrhea, chronic constipation) and participants who denied to sign consent form.

### 2.9 Study design

This trial was a double-blind, randomized, placebo-controlled trial conducted at Chiang Mai University Hospital, Chiang Mai, Thailand. The trial protocol was approved by the Research Ethics Committee of the Faculty of Medicine, Chiang Mai University (Ethical approval number 368/2562) and was registered at the Thai Clinical Trial Registry as identification number 20241117005. All participants provided written informed consent and were screened for inclusion and exclusion criteria. During screening, baseline data were collected from all participants, including medical history, physical examination, body composition analysis, and laboratory assessments. Laboratory tests included a complete blood count (CBC), fasting blood sugar (FBS), fasting insulin, HbA1c, lipid profile, blood urea nitrogen (BUN), creatinine (Cr), electrolytes, liver function tests (LFTs), thyroid-stimulating hormone (TSH), and urinalysis. Insulin resistance was estimated using the homeostasis model assessment of insulin resistance (HOMA-IR), calculated as: fasting glucose (mg/dL) × fasting insulin (mU/L)/405. In addition, urine pregnancy tests in childbearing-age women were collected. One week after screening, eligible participants were randomly assigned to receive either a 75-mL of CPE product or a placebo. A random allocation sequence using a computer-based problem. To ensure allocation concealment, sequentially numbered, opaque, sealed envelopes (SNOSE) were prepared by an independent statistician who was not involved in participant enrollment or outcome assessment. The envelopes were stored securely and opened only after eligibility had been confirmed and written informed consent had been obtained. All investigators, participants, and related medical staff were blinded to the intervention assignments throughout the study. Participants were required to shake and drink 75-mL of CPE product or placebo twice a day after breakfast and lunch every day for 24 weeks (at least 80% of the total product received and within 1 h after opening the bottle). At the randomization visit, the same laboratory data as the screening were collected, and a computed tomography (CT) abdomen was performed to evaluate visceral and subcutaneous fat. All participants were advised to restrict caloric intake to 1,200–1,500 kcal/day in women and 1,500–1,800 kcal/day in men (emphasized intake of a high-fiber, low-fat, and low-sugar diet) and were advised to exercise at least 30 min/day for 4 to 5 days a week. Participants were requested to record their 24-h food intake for 3 days a week, with 2 weekdays and 1 weekend, and to do physical activity records for the entire study period. Participants received standardized instructions and the same recording templates, and logs were reviewed at each visit for completeness and plausibility. Importantly, the intervention and placebo groups were monitored using identical procedures throughout the study, which helps minimize differential (between-group) reporting bias. Subjects had to return all food and physical activity record forms with every visit. After that, participants were evaluated at the end of weeks 4, 12, and 24 (all visits +/− 5 days), with a focus on assessment of adherence (asked participants to return the empty bottles and the remaining products), adverse events, body composition analysis, returning of food and physical activity record forms, assessment of satisfaction on the product and laboratory assessment. A CT abdomen was performed once again at the end of the study.

### 2.10 Statistical analysis

The previous study reported that participants with LDL-C ≥ 130 mg/dL would have a statistical error of 55% (37). Moreover, the CPE product could lower LDL-C in hyperlipidemic obese rats by approximately 28 to 56% (30). Thus, this study required 80 participants for enrollment to evaluate the effect of the CPE product on the primary outcome compared to placebo, assuming a dropout of 5%, a two-sided alpha level of 0.05, a delta of −36.0 (lower LDL-C 28%), and a power of more than 80%. Analysis was conducted using STATA 14.2 computer software (Stata Corporation, Texas, United States). Mean and standard deviation (SD) were used to present continuous variables. Categorical variables were presented as percentages. The Student t-test and the Wilcoxon rank sum test were used to determine the differences between the two independent samples of continuous variables with normal and non-normal distributions, respectively. The Chi-square test or Fisher’s exact test was used to determine the differences among the categorical variables, where appropriate. The transformation of data was carried out to make the data follow a normal distribution. For the continuous outcome variables, a Generalized Estimating Equation (GEE) was used. Time-by-covariate interaction and pretest of covariate data were considered at the time of analysis to assess whether each covariate’s impact on the outcome varied over time. Fisher’s least significant difference (LSD) was used for multiple comparisons. A two-sided *p*-value < 0.05 was determined to be statistically significant.

## 3 Results

### 3.1 The quality control of the CPE product

**Figure 2:**
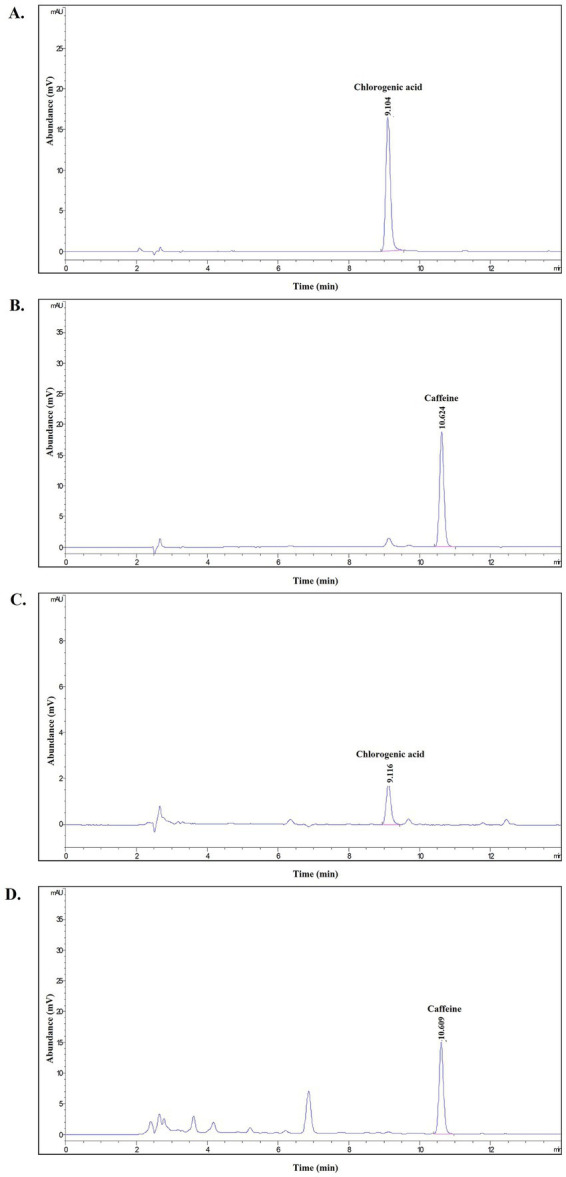
A representative HPLC chromatogram of **(A)** Chlorogenic acid standard, **(B)** caffeine standard, **(C)** *C. arabica* pulp extract (CPE) product for detecting chlorogenic acid, **(D)** *C. arabica* pulp extract (CPE) product for detecting caffeine at the absorption wavelength 280 nm.

To control the quality of the CPE product and placebo, the nutritional compositions, active compounds, and contamination testing were determined in both samples. As shown in Table 1, the main sources of energy in the CPE product and placebo were carbohydrates (88.00% vs. 94.12%), respectively, whereas cholesterol was undetectable in both samples. Moreover, the CPE product was composed of 1.15 g protein, 2.06 mg sodium, 15.86 mg calcium, and 0.72 mg iron and provided 58.05 kcal/100 mL. In comparison, the placebo contained 0.26 g protein, 4.83 mg sodium, 2.71 mg calcium, and less than 0.1 mg iron, with a total energy of 29.96 kcal/100 mL. In addition, the CPE product had significantly higher values of polyphenols at 9,270.00 ± 479.76 mg/L gallic acid, chlorogenic acid (CGA) at 4,505.67 ± 1,121 mg/L, and caffeine at 1,595.33 ± 209 mg/L than that of the placebo (82.47 ± 8.36, 0.65 ± 0.75, and 0.43 ± 0.13, respectively) as shown in Table 2. A representative HPLC chromatogram is shown in Figure 2. In addition, no microorganism growth exceeded the standard references in either sample, as indicated in Table 3. In addition, the radical scavenging activities values of CPE product against ABTS^+•^ and DPPH radicals at doses of 5–100% (v/v) were 8.26 ± 1.19 to 84.51 ± 2.95 and 43.98 ± 1.50 to 48.18 ± 1.9, respectively. This result suggests that the CPE product had a high antioxidant activity (Table 4).

**Table 1:** The nutritional compositions of CPE product and placebo.

Ingredients	Coffogenic drink (100 mL)			Placebo (100 mL)		
	Amount	Unit	%E	Amount	Unit	%E
Carbohydrates	12.77	g	88.00	7.05	g	94.13
Sugar	7.59	g		6.35	g	
Fiber	0.00	g		0.00	g	
Total Fat	0.27	g	4.11	0.08	g	2.40
Saturated fat	0.13	g		0.06	g	
Cholesterol	ND	mg		ND	mg	
Protein	1.15	g	7.89	0.26	g	3.47
Sodium	2.06	mg	–	4.83	mg	–
Calcium	15.86	mg	–	2.71	mg	–
Iron	0.72	mg	–	>0.1	mg	–
Moisture	83.14	g	-	–	g	–
Ash	2.68	g	–	0.05	g	–
Total	100.00	g	100	100.00	g	100
Kcal/100 mL	58.05 kcal/100 mL			29.96 kcal/100 mL		

ND, not detectable.

Energy per gram (kcal/g): carbohydrate = 4, fat = 9, protein = 4, ND, not detected.

**Table 2:** The quantity of polyphenols, chlorogenic acid, and caffeine of CPE product and placebo.

Compounds	Products		Unit	LOQ	LOD	Reference method
	Coffogenic drink	Placebo				
Polyphenols	9,270.00 ± 479.80	82.47 ± 8.36^*^	mg/L gallic acid	–	–	UV–Vis Spectrophotometry based on J. Food Chemistry, 92(2005) 491–497
Chlorogenic acid	4,303.67 ± 1,121	0.65 ± 0.75^*^	mg/L	–	–	HPLC
Caffeine	1,595.33 ± 209	0.43 ± 0.13^*^	mg/L	–	–	HPLC

LOQ, limit of quantitation; LOD, limit of detection. Values shown are mean ± SD (*n* = 3), *p < 0.05 vs. Coffogenic drink.

**Table 3:** The contamination testing of CPE product and placebo.

Test	Result		Unit	LOD	Reference method
	CPE product	Placebo			
*Bacillus cereus*	<1	< 1	CFU/mL	–	FDA BAM Online,2001; update 2012 (Chapter 14)
*Clostridium perfringens*	<1	< 1	CFU/mL	–	FDA BAM Online,2001 (Chapter 16)
Coliform	<1.1	< 1.1	MPN/100 mL	–	Standard Methods for the Examination of Water and Wastewater APHA, AWWA, WEF, 23rd Edition, 2017, Part 9221
*Escherichia coli*	Not detected	Not detected	In 100 mL	–	Standard Methods for the Examination of Water and Wastewater APHA, AWWA, WEF, 23rd Edition, 2017, Part 9221
*Salmonella* spp.	Not detected	Not detected	In 25 mL	–	ISO 6579-1:2017 (e)
*Staphylococcus aureus*	Not detected	Not detected	In 0.1 mL	–	FDA BAM Online,2001; update 2016 (Chapter 12)
Yeast and Molds	< 1	< 1	CFU/mL	–	FDA BAM Online,2001 (Chapter 18)

LOD, limit of detection.

**Table 4:** Radical scavenging activities of CPE product and its placebo.

Compound	Concentration	DPPH	ABTS
	% (V/V)	% inhibition	% inhibition
Placebo	5	0.23 ± 0.40	0.34 ± 0.22
	10	1.52 ± 0.47	0.68 ± 0.48
	25	2.08 ± 0.58	1.06 ± 0.67
	50	3.58 ± 1.61	1.40 ± 1.01
	75	4.33 ± 1.39	1.88 ± 1.27
	100	5.10 ± 0.82	4.30 ± 4.16
CPE product	5	43.98 ± 2.99	8.26 ± 3.40
	10	48.18 ± 3.90	25.54 ± 9.07
	25	>100	62.76 ± 11.51
	50	>100	79.56 ± 10.95
	75	>100	82.78 ± 6.68
	100	>100	84.51 ± 5.38
Vit C	5	71.67 ± 2.27	18.65 ± 11.74
	10	83.13 ± 1.33	42.43 ± 15.43
	25	85.45 ± 0.96	92.23 ± 6.25
	50	85.87 ± 0.22	92.27 ± 6.19
	75	86.07 ± 0.35	92.32 ± 6.18
	100	86.94 ± 0.21	92.35 ± 6.12

>100; out of range (*N* = 5 ± SD).

### 3.2 Changes in cholesterol micelle properties

**Figure 3:**
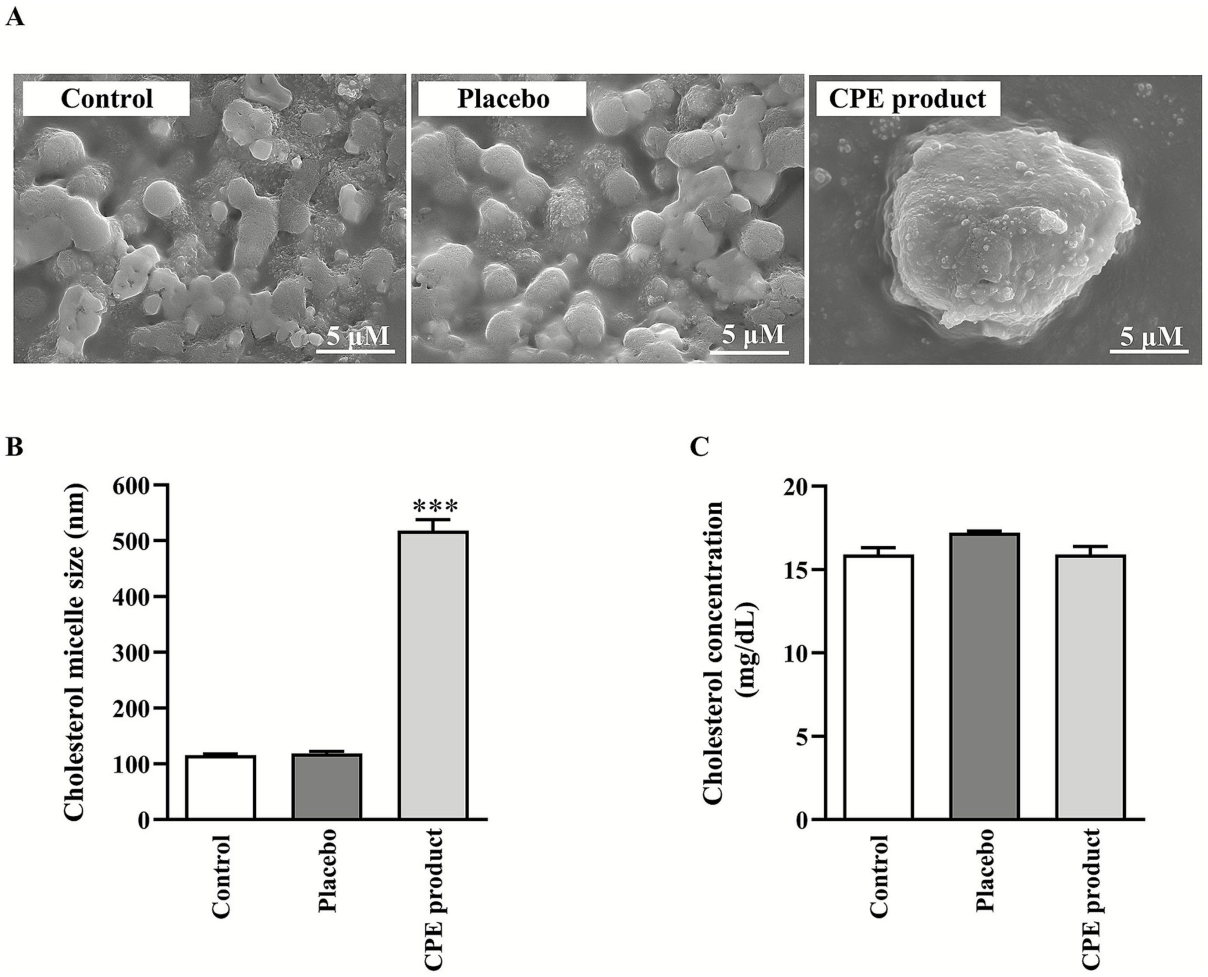
Effect of *C. arabica* pulp extract (CPE) product on **(A)** scanning electron microscope (SEM) images of cholesterol micelles, **(B)** micellar cholesterol particle size, and **(C)** intermicellar cholesterol levels. Values shown are mean ± SD (*n* = 5), ***p < 0.001.

As expected, the CPE product increased the mixed micelle particle size in SEM images compared to the control and placebo, as shown in the cholesterol micelle morphology (Figure 3A) and size (Figure 3B). However, the CPE product did not affect cholesterol solubility, as did the placebo (Figure 3C). As such, the CPE product possibly affects cholesterol micelle particle size, which might be associated with cholesterol-related outcomes in the subject.

### 3.3 Changes in bile acid binding capacity

To clarify the effect of CPE product on bile acid binding ability, the affinity of CPE product for primary bile acid (taurocholic acid) and secondary bile acids (taurodeoxycholate and glycodeoxycholate) was determined. As shown in Table 5, the CPE product significantly bound to only glycodeoxycholate. On the other hand, the placebo significantly bound to taurocholic acid and taurodeoxycholate. Therefore, the CPE product showed a strong affinity for secondary bile acid binding, resulting in impaired mixed micelle formation.

**Table 5:** Effect of CPE product on bile acid binding capacity *in vitro*.

Samples	Concentration	Bile acids (%)		
		Taurocholic acid	Glycodeoxycholate	Taurodeoxycholate
Control	–	0.00 ± 0.00	0.00 ± 0.00	0.00 ± 0.00
CPE product	5% (v/v)	0.00 ± 0.00	207.42 ± 85.06	0.00 ± 0.00
Placebo	5% (v/v)	194.68 ± 70.88	201.25 ± 71.58	362.81 ± 66.19
Cholestyramine	5% (w/v)	70.96 ± 67.08	113.46 ± 22.00	203.77 ± 52.07

### 3.4 Baseline characteristics

**Figure 4:**
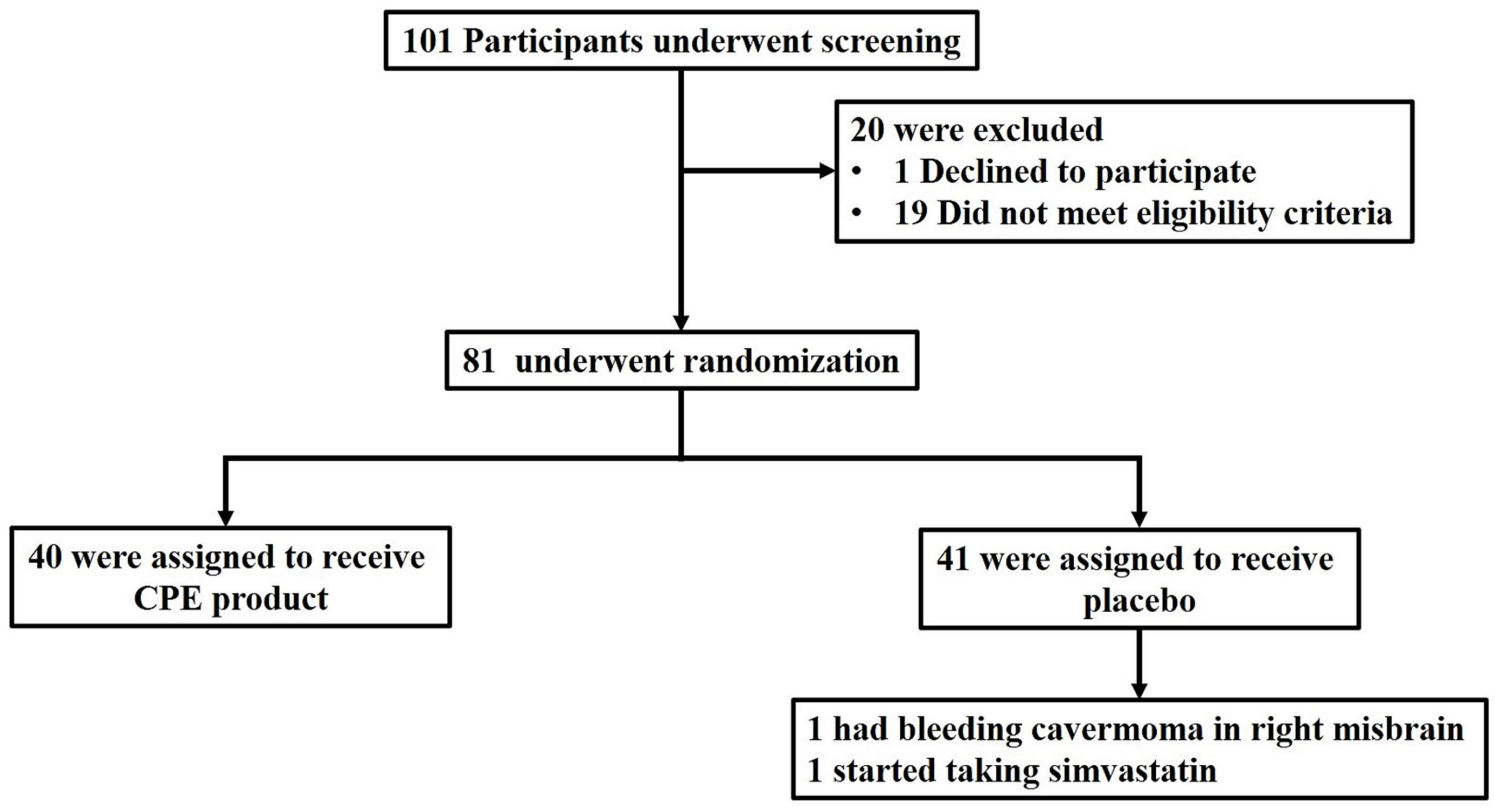
Diagrammatic synopsis of the participant screening, enrollment, and follow-up process. The primary analysis was included for only the participants who had completed the trial at week 24. CPE product denotes *C. arabica* pulp extract (CPE) product.

One hundred and one participants were evaluated for eligibility, and 81 participants met the inclusion criteria (Figure 4). All eligible participants were randomly assigned to receive either a CPE product or a placebo for 6 months, from November 2019 through June 2020, at Chiang Mai University Hospital, Chiang Mai, Thailand. At baseline, two participants (one from each group) had been on statin, and one from the CPE product group had been on fibrate at a stable dose for > 8 weeks prior to participating in the study. The baseline characteristics were not significantly different between experimental groups (Table 6). Before week 4, one participant in the placebo group withdrew from the study due to a bleeding cavernoma. At week 12, another participant in the placebo group was excluded after initiating simvastatin during the trial.

**Table 6:** Baseline characteristics of the participants.

Baseline characteristics	CPE product (*N* = 40)	Placebo (*N* = 39)	*P*-value
Age-year*	42.5 ± 10.2	43.0 ± 9.8	0.87
Male sex – no. (%)	15 (37.5)	15 (38.5)	0.93
Current smoker – no. (%)	1 (2.5)	3 (7.7)	0.36
Alcohol drinking – no. (%)	15 (37.5)	14 (35.9)	0.88
Systolic blood pressure (mmHg)*	129.1 ± 11.2	125.9 ± 14.2	0.26
Diastolic blood pressure (mmHg)*	81.4 ± 9.2	83.7 ± 7.7	0.22
Anthropometry			
Body weight (kg)*	77.9 ± 14.7	79.6 ± 14.4	0.61
BMI (kg/m^2^)*§	29.3 ± 4.0	30.0 ± 3.9	0.44
Waist circumference (cm)*	96.1 ± 9.3	97.8 ± 9.3	0.43
Fat mass (%)*	37.9 ± 7.7	37.8 ± 8.4	0.93
Lean body mass (%)^†^	47.3 (44.4, 50.3)	48.4 (45.2, 51.9)	0.61
Visceral fat area (cm^2^)^†^	105.31 (92.15, 120.36)	124.15 (110.24, 139.81)	0.0668
Lipid profiles			
LDL-C level (mg/dL)^†^	167.6 (159.3, 176.3)	177.0 (169.0, 185.4)	0.033
Total cholesterol (mg/dL)*	222.4 ± 33.2	235.0 ± 30.3	0.083
HDL-C level (mg/dL)^†^	53.7 (50.5, 57.1)	50.8 (47.5, 54.3)	0.217
Triglyceride (mg/dL)^†^	112.3 (95.3, 132.3)	140.9 (120.3, 165.1)	0.13
Metabolic parameters			
FBS (mg/dL)*	89.2 ± 11.0	91.1 ± 8.6	0.396
HbA1c (%)*	5.6 ± 0.4	5.5 ± 0.4	0.727
Insulin (μlU/mL)^†^	12.63 (10.58–15.08)	14.64 (11.97–17.90)	0.268
HOMA-IR (μU/mL)^†^	2.76 (2.27–3.34)	3.27 (2.64–4.05)	0.232

Data from the screening visit.

*Normally distributed data are presented as mean ± SD.

^†^Non-normally distributed data were log-transformed and are presented as geometric means (95% CI).

^§^The body-mass index is the weight in kilograms divided by the square of the height in meters.

### 3.5 Changes in lipid profiles

**Figure 5:**
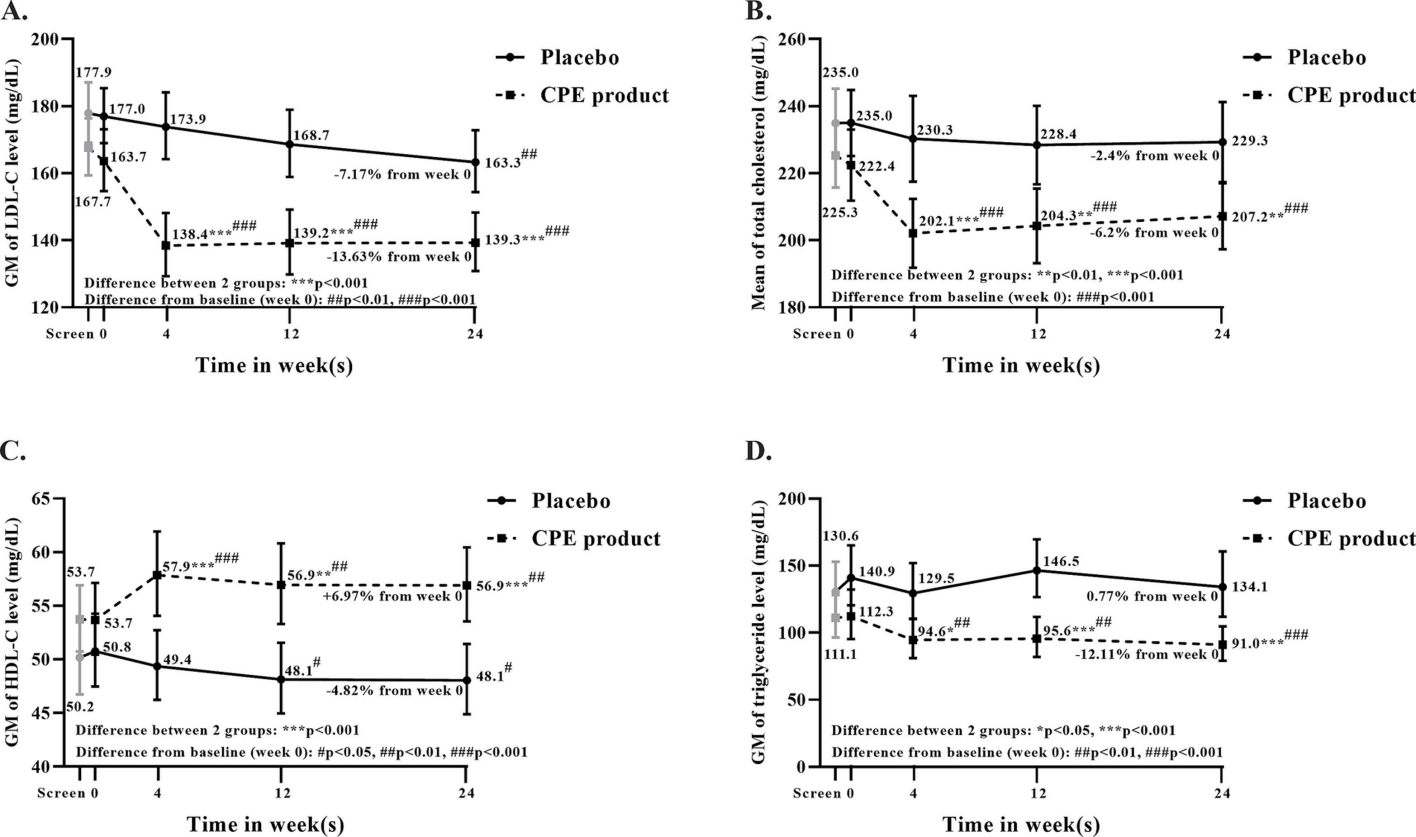
Effect of *C. arabica* pulp extract (CPE) product and placebo on lipid profiles compared between groups and from baseline to week 24. **(A)** Geometric mean of LDL-C levels, **(B)** mean of total cholesterol, **(C)** Geometric mean of HDL-C levels, and **(D)** Geometric mean of triglyceride levels. Data are represented as mean ± SD. *p < 0.05, **p < 0.01, ***p < 0.001 vs. placebo, ^#^*p* < 0.05, ^##^*p* < 0.01, ^##^*p* < 0.001 vs. baseline (week 0).

The percentage reduction of LDL-C, TG, and total cholesterol (TC) levels in the CPE product group at week 24 was 14.9, 18.9, and 8.0%, respectively, whereas HDL-C levels increased by 6.0% (*p* < 0.01 for all parameters compared to baseline). In the placebo group, TC and TG levels did not significantly change from baseline to week 24, but HDL-C and LDL-C levels significantly decreased by 5.3% and by 7.7%, respectively. CPE product significantly reduced TC (*p* = 0.005), LDL-C (*p* < 0.0001), and TG level (*p* < 0.0001) but increased HDL-C level (*p* < 0.0001) compared to the placebo group at week 24 (Table 7 and Figure 5). The percent changes in TC, LDL-C, and HDL-C were significantly greater in the CPE product group than those in the placebo groups at all visits in 4, 12, and 24 weeks. Whereas the percent change in TG level was significantly greater in the CPE product group than that of the placebo group in weeks 12 and 24 (Figure 5).

**Table 7:** The comparison of the lipid profiles in serum samples from participants supplemented with CPE product and the placebo group at baseline and week 24.

Lipid profiles	CPE product (*N* = 40)	Placebo (*N* = 39)	*P*-value between groups at the end of week 24
LDL-C (mg/dL)^†^			
At baseline	163.7 (154.7, 173.1)	177.0 (167.0, 185.4)	<0.0001
End of 24 week	139.3 (130.9, 148.3)	163.3 (154.4, 172.8)	
% change	−14.9	−7.7	
*P*-value from baseline	<0.0001	0.002	
Total cholesterol (mg/dL)*			
At baseline	225.3 ± 29.8	235.0 ± 31.7	0.005
End of 24 week	207.2 ± 30.7	229.3 ± 36.8	
% change	−8.0	−2.4	
*P*-value from baseline	<0.0001	0.127	
Triglyceride (mg/dL)^†^			
At baseline	112.3 (95.3, 132.3)	140.9 (120.3, 165.1)	<0.0001
End of 24 week	91.0 (79.1, 104.8)	134.1 (111.9, 160.7)	
% change	−18.9	−4.8	
*p*-value from baseline	<0.0001	0.364	
HDL-C (mg/dL)^†^			
At baseline	53.7 (50.5, 57.1)	50.8 (47.5, 54.3)	<0.0001
End of 24 week	56.9 (53.6, 60.5)	48.1 (44.9, 51.5)	
% change	+6.0	−5.3	
*p*-value from baseline	0.008	0.013	

Analyzed by using Generalized Estimating Equation (GEE) adjusted by data at screening visit.

*Normally distributed data are presented as mean ± SD.

^†^Non-normally distributed data were log-transformed and are presented as geometric means (95% CI).

After adjustment for screening lipid values, the CPE group showed greater reductions in TC, TG, and LDL-C at week 24 than the placebo group (−6.3% vs. −2.4%, *p* = 0.048; −12.5% vs. +1.17%, *p* = 0.078; and −13.7% vs. −7.1%, *p* = 0.01, respectively). HDL-C increased in the CPE group but decreased in the placebo group at week 24 after adjustment. The percent reductions in TC and LDL-C in response to the CPE product were significantly greater in female than in male participants (*p* = 0.004 and *p* = 0.007, respectively), whereas the percent increase in HDL-C was significantly greater in males than in females (*p* = 0.048). In contrast, the percent change in serum TG did not differ between males and females after baseline adjustment (*p* = 0.789, Supplementary Table S1). The percent reduction in LDL-C associated with the CPE product was significantly greater in older participants (≥45 years) compared with younger participants (<45 years; *p* = 0.008). No significant age-related differences were observed for percentage changes in TC, TG, or HDL-C after baseline adjustment (*p* = 0.146, 0.918, and 0.874, respectively; Supplementary Table S2).

### 3.6 Changes in proinflammatory markers

**Figure 6:**
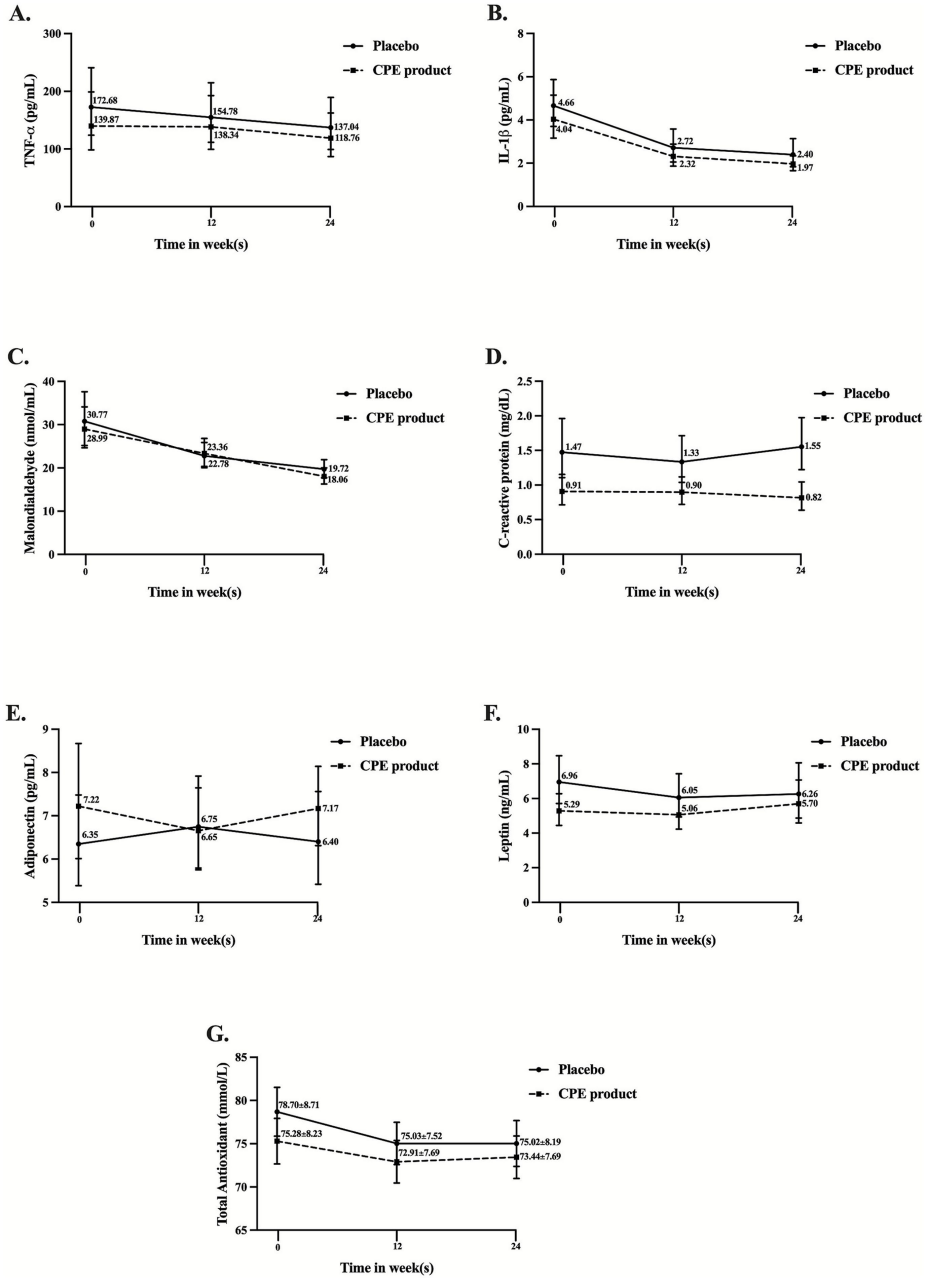
Effect of *C. arabica* pulp extract (CPE) product and placebo on inflammatory markers compared between groups and from baseline to week 24. **(A)** TNF-α, **(B)** IL-1β, **(C)** malondialdehyde, **(D)** C-reactive protein, **(E)** adiponectin, **(F)** leptin, and **(G)** total antioxidant levels. Data are represented as mean ± SD.

The levels of tumor necrosis factor-α (TNF-α; Figure 6A), interleukin-1β (IL-1β; Figure 6B), and malondialdehyde (MDA; Figure 6C) significantly decrease in both the CPE-treated group and the placebo over time, with no intergroup differences. C-reactive protein (CRP; Figure 6D) decreased over time in the CPE group, whereas it increased in the placebo group, resulting in a significant difference between groups at the study endpoint. Moreover, adiponectin (Figure 6E), leptin (Figure 6F), and total antioxidant (Figure 6G) remained unchanged between groups and over time, suggesting that 6 months of CPE supplementation had a slight effect on inflammatory status.

### 3.7 Changes in the secondary outcomes

Participants in the CPE product group significantly decreased in BW by 0.5 kg, BMI by 0.17 kg/m^2^, and waist circumference decreased by 1.96 cm at week 24 (*p* < 0.05 for all parameters compared to the baseline and compared between groups) whereas no change in other body composition in the CPE product group and no significant change in all body composition in the placebo group from the baseline. FBS was maintained in the CPE product group but increased in the placebo group. Therefore, there was a significant difference between groups at the end of week 24 (*p* = 0.0035). HbA1c and insulin levels did not change significantly in either group from the baseline and were not significantly different between groups at week 24. However, HOMA-IR, which reflects insulin resistance, was decreased significantly more in the CPE product group compared to the placebo group (*p* = 0.0276) (Table 8). Furthermore, data from CT abdomen demonstrated that there was no significant difference in all fat areas, visceral fat areas, subcutaneous fat areas, and girth between the CPE product and placebo groups (Supplementary Table S3).

**Table 8:** The changes in the secondary outcomes between groups and from baseline to week 24.

Outcome	CPE product (*N* = 40)	Placebo (*N* = 39)	*P*-value between groups
Body weight (kg)* [% change from baseline]	77.3 ± 15.4 [−0.7]	80.0 ± 14.0 [+0.5]	0.0047
BMI (kg/m^2^)* [% change from baseline]	29.1 ± 4.3 [−0.8]	30.2 ± 4.0 [+0.7]	0.0043
Fat mass (%)* [% change from baseline]	36.4 ± 8.5 [−4.0]	37.4 ± 8.9 [−0.9]	0.2093
Waist circumference (cm)* [% change from baseline]	94.1 ± 9.2 [−2.1]	96.9 ± 10.0 [−0.9]	0.0023
FBS (mg/dL)* (% change from baseline)	89.7 ± 9.40 [+ 0.6]	94.0 ± 8.8 [+3.2]	0.0035
HbA1C (%)* (% change from baseline)	5.6 ± 0.5 [+0.4]	5.6 ± 0.4 [+0.9]	0.3321
Insulin (μIU/mL)^†^ (% change from baseline)	10.6 (8.7, 12.8) [−16.5]	14.1 (11.3, 17.6) [−3.6]	0.0714
HOMA-lR (μU/mL)^†^ [% change from baseline]	2.3 (1.9, 2.9) [−15.6]	3.3 (2.6, 4.1) [−0.6]	0.0276

*Plus-minus value are means ± SD.

^†^Number in parentheses are presented by Geometric mean and 95% CI.

### 3.8 The three-day food record and weekly physical activity record

Participants in the CPE product groups consumed significantly more calories, protein, and fat than those in the placebo groups at the end of the study, without significant differences over time. The CPE product groups consumed an average of 1,429.4 (1,383.2, 1,477.2) kcal/d, 56.9 (55.6, 58.1) g of protein a day, and 45.2 (43.6, 47.0) g of fat a day. Whereas the placebo group consumed an average of 1,334.9 (1,291.2, 1,380.1) kcal/d, 49.8 (48.7, 50.9) g of protein a day, and 39.7 (38.2, 41.2) g of fat a day (*p* = 0.0042, < 0.00001 and < 0.00001, respectively). There was no significant difference in carbohydrate intake between the CPE product and the placebo groups (Supplementary Figures S1A–D).

Physical activity is calculated from 0.0175 × body weight (Kg) × duration of activity × Metabolic Equivalent (MET). The duration of activity in a week is equal to the duration of each activity in one time multiplied by the times in a week. Total physical activity was not significantly different between the 2 groups, with 8,166.7 (7,306.4, 9,128.4) kcal/week in the CPE product group and 8,262.7 (7,381.7, 9,248.8) kcal/week in the placebo group. However, both groups had decreased their physical activity over time (*p* = 0.0325) (Supplementary Figure S1E).

### 3.9 Product satisfaction scores

Participants were satisfied with the placebo product in the 70% range, whereas it was 56–67% in the CPE product group. The satisfaction rate was significantly greater in the placebo than in the CPE product at week 4 (*p* < 0.0001), week 12 (*p* < 0.0001), and end of week 24 (*p* = 0.009). Nonetheless, the satisfaction rate of the CPE product gradually increased significantly from week 4 to week 12 and then week 24 (*p* < 0.0001 and *p* = 0.003, respectively) (Supplementary Figure S2).

### 3.10 Safety data

Regarding biochemical data, there was no significant difference in complete blood count, BUN, creatinine, electrolytes, serum calcium, magnesium, phosphorus, and TSH. However, within-group analysis of the alkaline phosphatase level was significantly lower in the CPE product group at the end of the experiment (at the baseline = 69.3 ± 19.2 μ/L and at week 24 = 61.6 ± 16.64 μ/L, *p* < 0.00001) and significant between groups at week 24 (*p* < 0.00001).

Adverse events were found in total, 12 from the CPE product group and 4 from the placebo group, such as bloating, constipation, diarrhea, frequent bowel movements, nausea, fatigue, and palpitation (Table 9). Notwithstanding, all adverse events from the CPE product were mild and transient and were not significantly different from those in the placebo group. No participants discontinued the CPE product or the placebo due to adverse events. No serious adverse events occurred.

**Table 9:** Adverse events during the 24-week treatment period.

Adverse events	CPE product (*N* = 40)	Placebo (*N* = 39)	*P*-value
Any adverse event	12	4	0.05
Bloating	4	0	0.51
Constipation	1	1	0.45
Diarrhea	1	1	1.000
Dry throat (%)	0	1	0.25
Excessive sweating	0	1	0.25
Fatigue	1	0	1.000
Frequent bowel movement	1	0	1.000
Nausea	1	0	1.000
Insomnia	1	0	1.000
Palpitation (%)	2	0	1.000

## 4 Discussion

This clinical study has unveiled the potential health benefits of the CPE enriched in polyphenols and the CGA product. Not only does it reduce LDL-C, TC, and TG levels, but it also has additional favorable effects on HDL-C levels. This clinical study has unveiled the potential health benefits of the CPE enriched in polyphenols and the CGA product. Not only does it reduce LDL-C, TC, and TG levels, but it also has additional favorable effects on HDL-C levels. The reductions in LDL-C (and the concomitant decrease in HDL-C) in the placebo group could be explained by lifestyle modification (diet and exercise), which is recommended for all participants at the beginning. A low-fat diet could lower both LDL-C and HDL-C, especially over short-to-intermediate follow-up. Nevertheless, this could be explained by regression to the mean and biological variability. Lipid measures show intra-individual variability, and participants often enroll when values are relatively elevated. Therefore, some downward shift in repeat testing can occur in the placebo group due to regression to the mean or short-term behavioral changes. Importantly, our primary interpretation is based on between-group comparisons rather than within-group changes alone. We used baseline-adjusted analyses (and repeated-measures models across visits, where applicable) to reduce the influence of regression to the mean. Additionally, participant-reported diet and physical activity logs were reviewed throughout follow-up; while these measures are imperfect, no clear evidence suggested differential lifestyle changes sufficient to explain the larger improvements observed in the intervention group. While statistically significant reductions in body weight, BMI, waist circumference, and HOMA-IR were observed, the magnitude of these changes was modest and is unlikely to translate into clinically meaningful benefits. Therefore, CT-based abdominal measurements may not have been sufficiently sensitive to detect corresponding small changes in visceral or subcutaneous fat areas, which may explain the lack of statistically significant differences between groups. Accordingly, these results should be interpreted as demonstrating statistical rather than clear clinical significance.

Regarding the placebo comparison, both the CPE product and placebo were administered in addition to participants’ habitual diets, which were maintained throughout the study period. Participants in both groups received identical dietary advice and lifestyle recommendations, and no specific dietary intervention was imposed. Thus, the primary difference between groups was the assigned study product, allowing for an appropriate placebo-controlled comparison. With respect to the polyphenol and caffeine content, the higher levels in the CPE product reflect its active composition and intended mechanism of action. The daily caffeine intake from the CPE product was within the range generally regarded as safe for healthy adults and below the maximum level of 400 mg/day considered safe for healthy adults according to the European Food Safety Authority and other international guidelines (38). No caffeine-related adverse events or safety concerns were observed during the study, and overall tolerability was comparable between the CPE and placebo groups.

Physical activity was encouraged at the beginning of the study, and both groups initially increased their activity levels. However, by week 24, most participants had returned to their usual physical activity habits, with no significant differences observed between groups. This change may have influenced lipid and metabolic parameters when compared with baseline values; however, because physical activity patterns were similar in both groups at the end of the study, it is unlikely to have affected the between-group comparisons. Therefore, any potential changes attributable to initiating lifestyle modification should have affected both groups similarly, and the between-group differences are most plausibly related to the intervention rather than unequal exercise guidance. Participant satisfaction scores were consistently lower in the CPE group than in the placebo group. However, adherence rates did not differ significantly between groups during the study period. While lower satisfaction may have implications for long-term adherence and real-world applicability, this did not appear to affect compliance within the controlled trial setting.

Alkaline phosphatase levels were significantly lower in the CPE group at week 24 compared with baseline and with the placebo group. However, the absolute changes were small, all mean values remained within the normal reference range, and there were no parallel abnormalities in other liver enzymes or clinical symptoms. We therefore interpret this as a statistically significant but clinically modest change, indicating that the CPE product was not hepatotoxic and that the isolated reduction in alkaline phosphatase is unlikely to have major clinical implications. In our study, 12 adverse events were reported in the CPE group and 4 in the placebo group; this difference was not statistically significant, and all events were mild, self-limited, and did not lead to treatment discontinuation. The events were mainly gastrointestinal in nature and were not accompanied by clinically relevant changes in laboratory parameters or vital signs. Given the absence of serious or persistent events, the lack of a consistent pattern suggesting organ toxicity, and the overall favorable biochemical profile, we interpret the higher number of mild adverse events in the CPE group as indicating acceptable tolerability rather than a major safety concern.

Our previous *in vivo* study also supports these findings, reporting a significant lipid-lowering effect of CGA rich in CPE after 12 weeks of supplementation in obese rats with induced hepatic steatosis. The possible mechanisms of CPE include inhibiting intestinal cholesterol absorption, interfering with cholesterol micellar complex formation, and increasing the hepatic lipolysis genes (29, 30). Consistent with our pre-clinical investigations, this study also found that the changes of physicochemical properties of the cholesterol micelles of polyphenols and CGA rich in CPE included enlargement of the cholesterol micelle size and interference with the affinity for primary bile acid binding. This could certainly display the mechanistic influence on the reduction of LDL-C, TG, and TC in the subjects who received the CPE product for 6 months. To support this notion, cholesterol micelle formation from fat digestion has been known to be an essential factor in determining human intestinal lipid absorption (39).

Similarly, a recent study demonstrated that supplementing 500 mg of green coffee bean extract rich in CGA for 12 weeks in healthy overweight subjects decreased LDL-C, TC, and TG levels and increased HDL-C levels. Furthermore, CGA also reduced BW, BMI, body fat (%), fat mass, and lean mass compared to a placebo group (40). In addition, the visceral fat area, total abdominal fat area, BW, and waist circumference significantly decreased in the coffee-enriched in CGA-treated overweight subjects compared with the placebo subjects (41). The mechanism underlying the lipid-lowering effect of CGA is elevated energy expenditure and fat oxidation (42). Our previous study also showed that CPE derived from naturally inhibited cholesterol micelle transport mediated by Niemann-Pick C1-like 1 (NPC1L1) in Caco-2 cells and rat jejunal absorptive epithelial cells, similar to ezetimibe, a prescription drug (29). Ezetimibe has shown the inhibition of uptake of dietary and biliary cholesterol, subsequently reducing LDL-C and non-HDL-C levels (43), and ezetimibe interferes with cholesterol trafficking from the plasma membrane to the endoplasmic reticulum in Caco-2 cells (44). Ezetimibe 10 mg daily was well tolerated and reduced LDL-C levels by approximately 17% (45), which was approximately the same proportion as the CPE product reduced in this study.

On the other hand, several studies have previously exhibited the effect of natural supplements on the change of lipid profiles with different mechanisms. Soluble Dietary Fiber (SDF), found in fiber-rich foods, has shown hypocholesterolemia benefits by reducing LDL-C and TC by approximately 5–10%. Potential mechanisms of SDF’s lipid-lowering effect include lowering overall energy intake because of its longer digestion time, increasing the rate of bile acid excretion and reducing bile acid reabsorption from the small intestine, and invoking the formation of short-chain fatty acids associated with reduced cholesterol synthesis (46). However, the lipid-lowering mechanism of SDF is different from CPE product. Additionally, intake of more than 2 g/day of plant sterol/ stanols is associated with additional and dose-dependent reductions in LDL-C (47). Besides, after 5 weeks of intervention, plant sterols in overweight participants had significantly lower LDL-C, HDL-C, and apolipoprotein B levels than in the control group (48). The possible mechanism is that the plant sterol ester competes with cholesterol for solubilization into a micelle, decreasing cholesterol absorption. A meta-analysis also demonstrated the effects of soy protein-containing isoflavones on the lipid profile. Soy protein containing isoflavones decreased LDL-C, TC, and TG by 5.25, 3.77, and 7.27%, respectively, and increased HDL-C by 3.03%. Mechanisms of soy isoflavones on lipid profile may be from isoflavones, which serve as a natural selective estrogen receptor modulator, and possibly from the effects on hepatic lipase. Soy protein peptide chains may also up-regulate LDL receptors (49). Likewise, after 8 weeks of consuming red yeast rice (RYR) extract supplements, LDL-C and TC levels were lowered by 22 and 15%, respectively. RYR contained monacolin K, which has a similar composition to lovastatin. Monocalin K can inhibit hydroxymethylglutaryl-coenzyme A (HMG-CoA) reductase, inhibiting liver cholesterol synthesis (50). Our recent study also demonstrated that CPE redistributed tight junction proteins, including occludin and zonula occludens-1, via a sirtuin-1-dependent pathway, leading to stabilization of the intestinal membrane barrier in the human intestinal epithelial-like T84 cell line (51). In fact, it has been known that obesity also impairs the tight junction barrier due to chronic low-grade inflammation in both rodents and humans (52, 53). Thus, besides blocking cholesterol absorption, CPE could potentially and mechanistically protect intestinal membrane integrity, leading to restoring systemic leakage inflammation, and delaying the progression of obesity complications.

## 5 Conclusion

The strengths of this study include the use of a locally sourced, natural, Thai-developed CPE product enriched in polyphenols and chlorogenic acid (CGA), which adds value to an agricultural by-product that would otherwise be discarded. Additional strengths include the randomized, double-blind, placebo-controlled design; the intermediate-term follow-up period (24 weeks); rigorous characterization of the intervention product; and the integration of mechanistic and clinical evidence. The intervention also demonstrated favorable safety and tolerability, and the study highlights an innovative approach to valorizing agricultural waste by converting it into a nutraceutical.

Key limitations include the relatively small sample size and the single-center design, which restrict the generalizability of the findings beyond Thai adults with hyperlipidemia and obesity. Although CPE produced statistically significant improvements in serum lipid profiles, the magnitude of effect was modest—approximately a 15% reduction in LDL-C—suggesting an effect size that is lower than that of low-intensity statins and more comparable to that of the cholesterol absorption inhibitor ezetimibe. An intention-to-treat analysis was not performed because only two participants in the placebo group discontinued the study. In addition, long-term cardiovascular outcomes were not evaluated; therefore, the potential role of CPE in cardiovascular risk reduction or prevention cannot be inferred from the present data. The dietary intake and physical activity, which were monitored by a 24-h dietary record and weekly physical activity record by participant self-report, may be at risk of recall bias, underreporting, and inter-individual variability.

In summary, 24-week consumption of the polyphenol- and chlorogenic acid (CGA)–rich CPE product was associated with favorable lipid profile changes in adults with hyperlipidemia and obesity, including reductions in LDL-C, total cholesterol, and triglycerides, along with an increase in HDL-C. However, the study had a small sample size and was exploratory in nature, as we included only Thai obese hyperlipidemia subjects. The observed lipid changes are smaller than those typically achieved with pharmacologic therapies, and high-risk patients should receive guideline-directed pharmacotherapy, with our product considered only as an adjunct. Larger studies are warranted to further characterize the safety profile of CPE. In addition, the lack of comparison with commonly consumed beverages limits the understanding of the antioxidant activity.

## Data Availability

The original contributions presented in the study are included in the article/Supplementary material, further inquiries can be directed to the corresponding author.
