# Supplementary material for: Potential lipid-lowering effects of Coffea arabica pulp extract product in hyperlipidemia-obese subjects: a randomized double-blind placebo-controlled trial
Source: Front Nutr. 2026 Mar 12;13:1755054. doi: 10.3389/fnut.2026.1755054 (PMC13017307; doi:10.3389/fnut.2026.1755054)
Supplement: Supplementary file 1 [file Data_Sheet_1.docx]

Supplementary Material

# Supplementary Table

**Table S1 Percent changes in lipid profiles associated with the CPE product at week 24 after baseline adjustment, stratified by gender**

| % change | Male | Female | p-value |
| --- | --- | --- | --- |
| Total cholesterol | -1.2 (-5.6 to 3.1) | -9.2 (-12.6 to -5.9) | 0.004 |
| Triglyceride | -10.6 (-25.4 to 4.3) | -13.5 (-29.0 to -2.0) | 0.789 |
| LDL-C | -7.6 (-13.2 to -2.0) | -17.2 (-21.5 to -12.9) | 0.007 |
| HDL-C | 11.2 (6.0 to 16.5) | 4.6 (0.5 to 8.6) | 0.048 |

**Table S2 Percent changes in lipid profiles associated with the CPE product at week 24 after baseline adjustment, stratified by age groups**

| % change | Younger age (< 45) | Older age (≥ 45) | p-value |
| --- | --- | --- | --- |
| Total cholesterol | -4.6 (-8.1 to -5.5) | -8.7 (-12.9 to -4.5) | 0.146 |
| Triglyceride | -12.9 (-28.3 to 2.5) | -11.7 (-27.3 to 3.8) | 0.918 |
| LDL-C | -9.9 (-14.3 to -5.5) | -19.3 (-24.7 to -13.9) | 0.008 |
| HDL-C | 6.9 (2.8 to 11.0) | 7.4 (2.4 to 12.4) | 0.874 |

**Table S3 Compared visceral fat from CT abdomen between groups and from baseline to the end of week 24**

| **CT abdomen** | **CPE group** | **Placebo** | ***P*-value between groups at the end of week 24** |
| --- | --- | --- | --- |
| **All fat area (cm^3^)**   - Day 1 - End of week 24 | 391.0 (358.3, 426.7)  390.6 (353.2, 432.0) | 418.8 (383.2, 457.7)  426.6 (386.8, 470.6) | 0.459 |
| **Visceral fat area (cm^3^)**   - Day 1 - End of week 24 | 105.3 (92.2, 120.4)  104.3 (91.0, 119.5) | 124.2 (110.2, 139.8)  125.7 (110.9, 142.4) | 0.388 |
| **Subcutaneous fat area (cm^3^)**   - Day 1 - End of week 24 | 277.2 (250.6, 306.7)  278.3 (247.4, 312.9) | 286.0 (256.5, 319.0)  291.3 (257.1, 330.0) | 0.587 |
| **Girth (cm)**   - Day 1 - End of week 24 | 99.3 ± 8.8  99.8 ± 10.0 | 101.2 ± 9.6  103.3 ± 18.1 | 0.551 |

Analyzed by repeated Measures Mixed Model

Compared means with Fisher’s protected least-significant (LSD)

**Supplementary Figures**

**
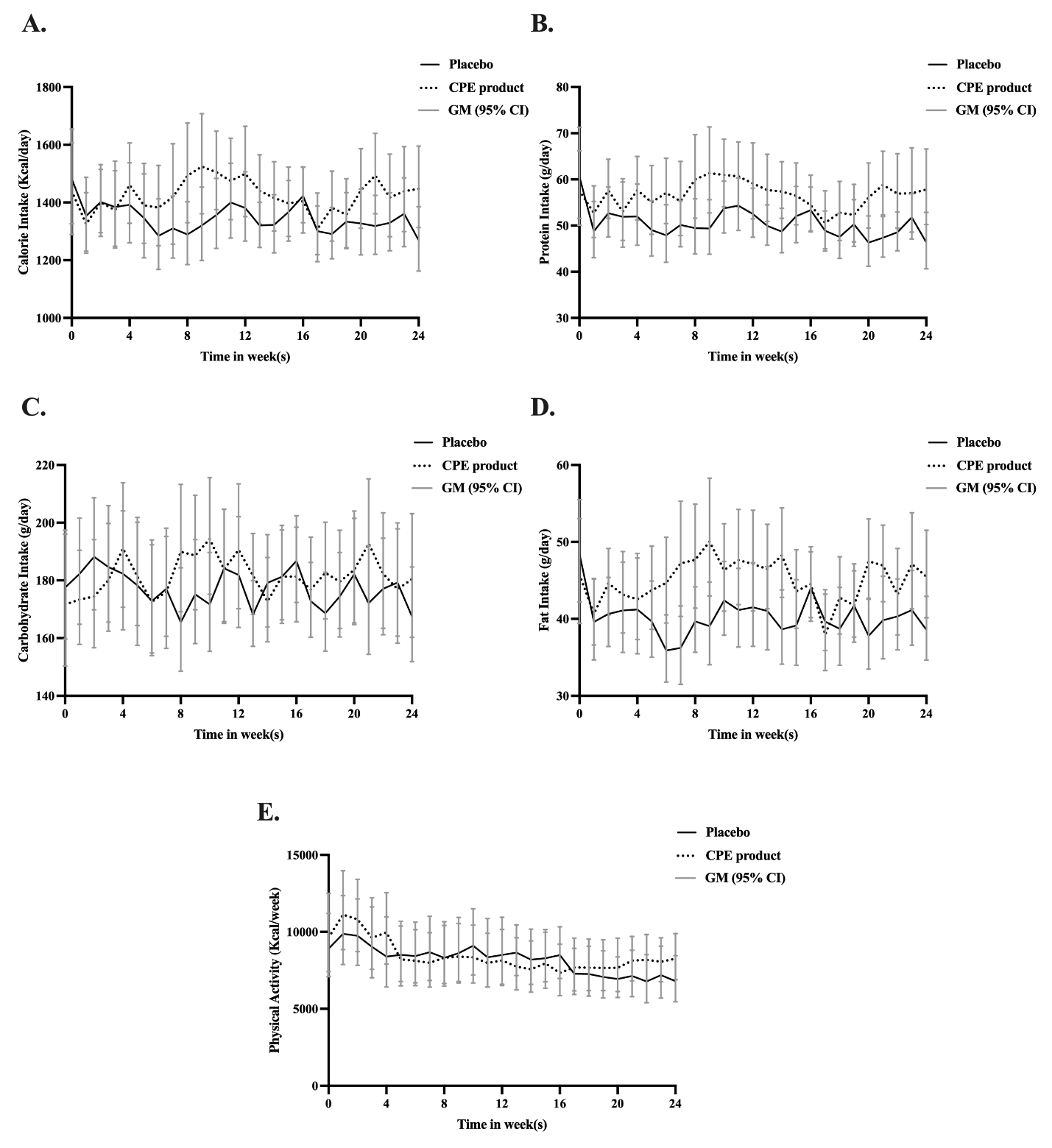
**

**Figure S1 Three-day food and exercise record**

**
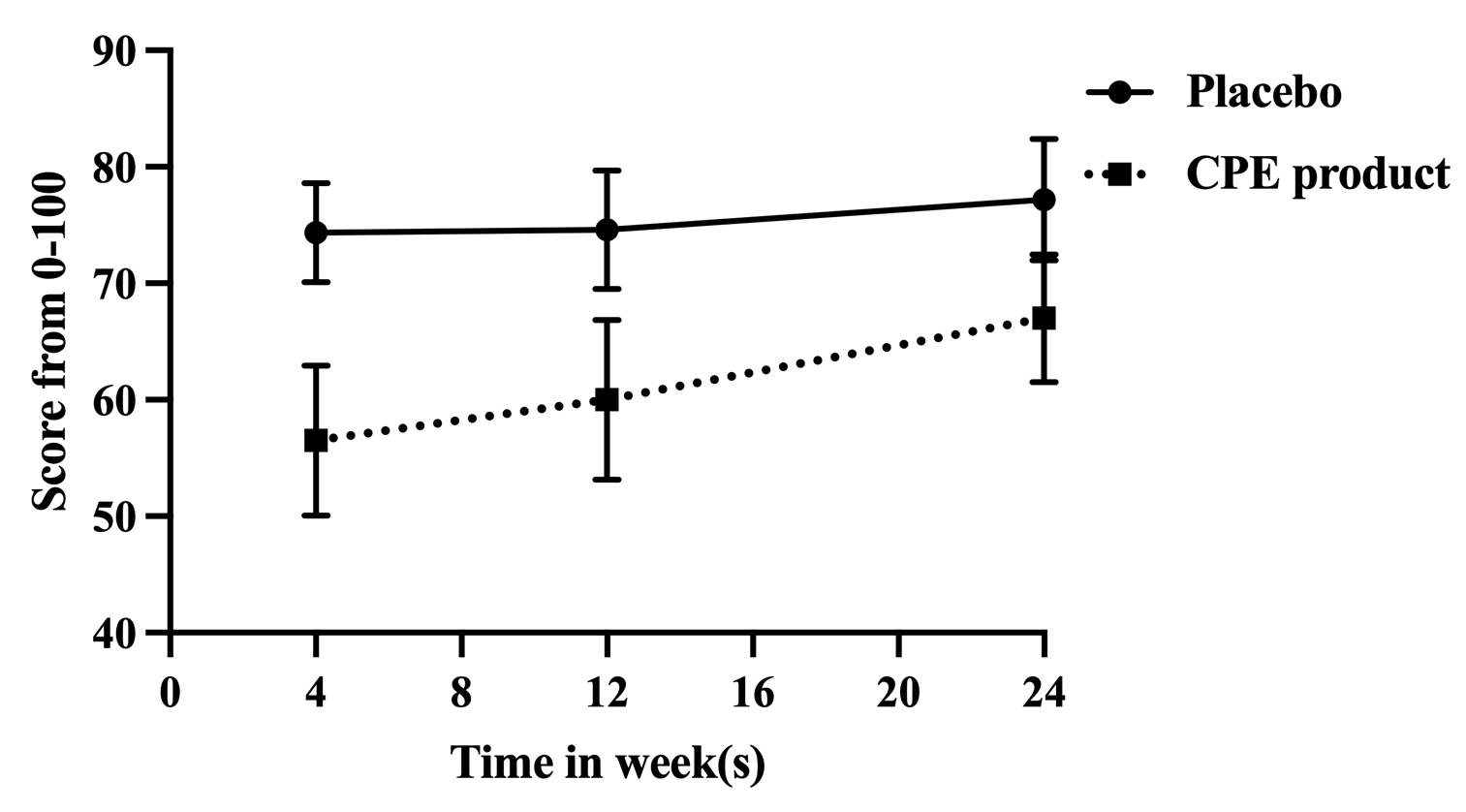
**

**Figure S2 Rate of satisfaction of the products**
